# The effects of causal and self-efficacy beliefs on help-seeking for people with depressive complaints: a quasi-experimental online study

**DOI:** 10.3389/fpsyt.2023.1232848

**Published:** 2023-11-30

**Authors:** Thomas McLaren, Lina-Jolien Peter, Samuel Tomczyk, Holger Muehlan, Georg Schomerus, Silke Schmidt

**Affiliations:** ^1^Institute of Psychology, University of Greifswald, Greifswald, Germany; ^2^Department of Psychiatry and Psychotherapy, University Hospital Leipzig, Leipzig, Saxony, Germany

**Keywords:** depression, help-seeking intervention, causal beliefs, self-efficacy beliefs, mental health stigmatisation, quasi-experimental online-study

## Abstract

**Background:**

Only approximately a third of people with depressive symptoms seek professional health care. Furthermore, people labelled as mentally ill may experience stigmatisation, which can impede help-seeking behaviour.

**Aim:**

To examine the effects of three vignette-based interventions endorsing biopsychosocial causal beliefs and strengthening self-efficacy on help-seeking intention and behaviour, as well as the predictive values of these variables and previous treatment experience.

**Method:**

A quasi-experimental online study utilising a fractioned factorial design was carried out. People were screened for depressive symptoms and their current treatment status. After baseline assessment, they were randomly allocated into one of 24 groups receiving a combination of interventional messages. Actual help-seeking behaviour was measured at follow-ups 3 and 6 months after baseline.

**Results:**

Altogether, *N* = 1,368 participants were included in the final analyses and *N* = 983 provided data on their help-seeking behaviour within 3 to 6 months after the baseline assessment. The intention to seek help from a general practitioner or a mental health professional was significantly influenced by the interventions. However, help-seeking behaviour was not influenced by the interventions. On a conceptual level, biopsychosocial causal beliefs (*β* = 0.09–0.23) and self-efficacy to seek help (*β* = 0.16–0.25) predicted help-seeking intention. There was a negative interaction effect of both self-efficacy beliefs on intention and behaviour, which changed depending on depression severity. In all models, the intention was the main predictor of actual behaviour. Treatment experience predicted both help-seeking intention and behaviour.

**Conclusion:**

Biopsychosocial causal beliefs and self-efficacy have a direct effect on help-seeking intention. Interventions should include information on how to actually seek help as a means to strengthen self-efficacy beliefs and simulate previous treatment experience. Further research is needed to investigate the respective interaction effects on intention and behaviour.

**Clinical Trial Registration:**

https://drks.de/search/de/trial/DRKS00023557, German Clinical Trials Register: DRKS00023557. Registered 11 December 2020. World Health Organization, Universal Trial Number: U1111–1264-9954. Registered 16 February 2021.

## 1 Background

Depression is a major burden in modern post-industrial countries (1). In Germany, only approximately a third of the people with depressive symptoms seek mental health care and usually with considerable delay after symptom onset (2). This issue is found in many countries with well-developed primary care, even when there is a specialised mental health care system (3). Furthermore, during the Covid-19 pandemic rates of depressive symptoms became more prevalent, exacerbating the issue (4).

Biddle et al. (5) defined help-seeking for psychological complaints as a social process whereby the focus lies on how symptoms are interpreted and managed by individuals and their communities. They argued that in a “cycle of avoidance” [(5), p. 988] the layperson will renegotiate what is normal so as to avoid the label of “illness” as long as possible and postpone seeking professional health care, wanting to manage the illness by themselves.

Avoiding the label of mental illness is understandable when considering that those labelled as mentally ill may experience stigmatisation for their mental illness (6, 7) and are often seen as dangerous, unpredictable, and unreliable (8). Mental health-related stigma is part of a network of beliefs deterring professional help-seeking behaviour. In a systematic review, Clement et al. (9) found that when all types of barriers are considered, anticipated stigma, especially regarding disclosure and confidentiality concerns, is the fourth highest barrier and has a negative effect on help-seeking (*d* = −0.27, (9), p. 15). Another study reported that the largest reported treatment-seeking barriers are wanting to handle the problem on one’s own and a low perceived need for care (10).

To overcome stigmatising attitudes and empower help-seeking, we suggest that it is essential to strengthen an individual’s biopsychosocial causal and self-efficacy beliefs. We have defined help-seeking as a process with four consecutive steps, from symptom awareness, over self-identifying as having a mental illness, to help-seeking intention and, finally, behaviour (11).

Depending on what a person believes to be the cause for their mental health complaints, they are more or less likely to seek help. For example, a biogenetic explanation will increase the likelihood that a person seeks help from a general practitioner (12) or a psychiatrist (13) compared to a psychosocial explanation. However, the biogenetic causal point of view is debated when considering its possible effect on stigmatising attitudes (14). On the one hand, biogenetic beliefs (e.g., heredity) have been found to reduce some aspects of stigma [e.g., (15)] because they imply that the person is not to blame for their illness. On the other hand, biogenetic beliefs facilitate an internal and stable illness attribution (14, 16). This essentialist attribution not only increases beliefs in dangerousness and differentness (17), it will also likely produce the idea that therapeutic help is not effective as the cause is assumed to be too stable to change, reducing help-seeking behaviour.

In contrast, a *balanced biopsychosocial model* from the perspective of vulnerability-stress research shows promising results both to counteract stigma (15, 17) and facilitate help-seeking intention (18). Such a model encompasses biogenetic as well as psychological and social causal beliefs and allows a person to regard the cause of their issues holistically and not just as the result of one (type of) cause (e.g., biomedical).

Causal beliefs can be seen as conceptually related to another important predictor for help-seeking behaviour: *agentic self-efficacy* (19). The *Attribution Theory* (20) with its three main attributional dimensions, namely, “stability,” “locus,” and “control,” implies this. For example, if a person believes a cause for their depression to be their “weak will” (i.e., stable, internal, and non-controllable), then they are also more likely to have lower self-efficacy beliefs and vice versa. Research on self-efficacy has demonstrated that people are more likely to engage in certain behaviours if they believe their efforts will be successful (19, 21). This has been shown for a variety of health behaviours (22–24). Additionally, findings support the notion that general self-efficacy is a universal construct yielding significant relations with other psychological constructs (25). Because help-seeking behaviour for mental illness is a complex form of psychosocial health behaviour, involving planning and execution, the role of self-efficacy has to be considered within this context (5, 26, 27).

Furthermore, self-efficacy should be considered domain specific (21) and will be operationalised as such in the current study. The somewhat complementary self-efficacy forms under observation pertain to *self-help* and *seeking professional help*. First, the specific form of *self-efficacy to self-help* was included in the study because previous research indicated that it could negatively influence the help-seeking process, specifically the intention to seek help (27). Furthermore, it is rarely examined separately from general self-efficacy. Only two studies were identified in which similar task-specific self-efficacy was investigated (28, 29).

Second, the specific form of *self-efficacy to seek professional help* was included because we suggest that it could help bridge the “intention-behaviour gap” (23). This “gap” describes the problem that people do not show help-seeking behaviour despite their intent to do so, even when they have positive attitudes towards help-seeking and few perceived barriers [e.g., (30, 31)]. One reason for this gap is mental health stigma, which is negatively associated with self-efficacy [e.g., (32)], wherefore it seems prudent to try and bolster self-efficacy beliefs, specifically concerning help-seeking behaviour, as a way to support health care utilisation.

In this context, *previous treatment experience* was identified as an important distal predictor of help-seeking, positively influencing self-identification as having a mental illness (15). However, what possible impact *satisfaction with previous treatment* has on help-seeking has not been considered in the context of different stigmatising attitudes, causal beliefs, and self-efficacy beliefs.

The aim of the current study is twofold and corresponds to the three primary research questions formulated in the study protocol (11). First, we analysed the effects of informational, vignette-based, and online-administered interventions, respectively, endorsing a *balanced biopsychosocial causal model of mental illness*, *self-efficacy to self-help*, and *self-efficacy to seek professional help*. Second, and in addition to the study protocol, we investigated the conceptual associations and predictive values of these variables on help-seeking intention and behaviour. Because of the conceptual relatedness between causal and self-efficacy beliefs, possible interactions were considered within the analyses. The respective research questions were:

1. Does a balanced biopsychosocial causal model of depression positively influence help-seeking intention?
2. Does a higher self-efficacy to self-help negatively influence help-seeking intention?
3. Does a higher self-efficacy to seek help positively influence help-seeking intention and does it make help-seeking behaviour more likely?
4. Is treatment experience positively associated with both self-efficacy beliefs, as well as help-seeking intention and behaviour? In addition, how is satisfaction with previous treatment associated with these variables?

## 2 Methods

### 2.1 Sample

Before data was acquired via the online panel “respondi AG”, the study was preregistered (German Clinical Trials Register: DRKS00023557). Information on the complete sampling procedure, general power analysis, online-panel information, as well as anticipated participant flow is documented in the study protocol (11).

Altogether, *N* = 10,348 people were screened for eligibility. Participants were included in the study if their PHQ-9 sum score was ≥8, i.e., at least mild depressive symptoms (33), and they were currently not in professional treatment. Of the screened participants, *N* = 2,132 were deemed eligible for the study. After the baseline assessment, they were subsequently invited to participate in the second part of the assessment 36 h later, which included the interventions.

After both baseline and intervention assessment, *N* = 1,751 participants remained within the study. Participants were excluded from data analyses if their PHQ-9 score was <8 at the intervention assessment (*n* = 362), due to disparate gender data between the study points (*n* = 12) and because they were of diverse gender (*n* = 9), leaving a sample size of *N* = 1,368 after the intervention allocation. After 3 and 6 months, participants were invited to participate in follow-up assessments in which help-seeking behaviour for their mental health complaints was assessed. After 3 months, *N* = 983 people participated in the follow-up, and after 6 months, *N* = 829 people participated in the last follow-up.

Participant allocation to the intervention groups, drop-outs, and reasons for exclusion are reported in Supplementary Figure S1.

### 2.2 Online intervention

During the second part of the study, i.e., 36 h after baseline, the participants were randomly allocated into one of 24 groups, following a fractioned factorial design. All groups received a vignette in which a fictional person described their depressive symptoms. After reading the vignette, most participants received a combination of up to five interventional messages, aimed at influencing different psychological beliefs, i.e., continuum beliefs, mental health literacy, causal beliefs, and self-efficacy beliefs. The last two psychological beliefs are the focus of this study.

For example, the text aiming at strengthening self-efficacy to seek help had the following key messages: “After a lot of back and forth, I decided to seek professional help. I knew it would be a challenge, but I wanted change and my friends encouraged me with their experiences. […] I wasn’t prepared for how long I had to wait for an appointment with the therapist but I stuck to my plan. When therapy started, I thought, ‘everything will get better’. However, some sessions were very tough! Still, I am happy I found my therapist and now I understand that depression is treatable.”

The 24 groups were systematically determined within a fractioned factorial design (34). What group received what combination of interventional messages can be seen in the supplementary material (Supplementary Table S2). For example, participants allocated to group number 1 received no further messages after reading the depression vignette, participants in group number 2 received two interventional messages (pertaining to self-efficacy beliefs), participants in group number 14 received two interventional messages (pertaining to causal beliefs and self-efficacy to self-help), and participants in group number 16 received all five interventional messages (including the three different messages on causal and self-efficacy beliefs). This experimental design allowed for the grouping of multiple groups so that they could be analysed together as one experimental group. Furthermore, interactional effects could be analysed when participants received more than one interventional message. After each interventional message, the participants were asked to answer a simple question as a means to prompt reflection (e.g., “What do you think about the decision to seek professional help?” after the interventional message pertaining to self-efficacy to seek help).

For in-depth information on the fractioned factorial intervention design, its pros and cons as well as all material verbatim used in this study, we refer to the study protocol and its supplementary material, which was published via Open Access (11).

The material was written after extensive research on the target constructs, including a systematic review of correlation and intervention studies on continuum beliefs and mental illness stigma (35), and refined after cognitive debriefings (36) with people with depression (*N* = 15). Some intervention elements, including self-efficacy to seek professional help, were administered both in either text or video form. For this study, the analyses were done with the text-based interventions. For extensive information on why video interventions were used and what aim this had, we refer to the study protocol (11).

### 2.3 Measures

Sociodemographic data, depressive symptoms (*PHQ-9*), causal beliefs, self-efficacy to self-help, self-efficacy to seek professional help, help-seeking intention, previous treatment experience and satisfaction, and different stigmatising attitudes were assessed at baseline. For a pre-post intervention comparison, the target variables were again assessed after the intervention 36 h later. All target variables as well as help-seeking behaviour were assessed during the 3-and 6-month follow-ups. Reliability estimations in terms of internal consistency coefficients stated below are based on this study sample.

*Depression severity* was measured with the *PHQ-9* (33). Participants were asked to rate how often they had been bothered by complaints (e.g., “little interest or pleasure in doing things”) over the past 2 weeks, with the response options as follows: 0 = “not at all,” 1 = “several days,” 2 = “more than half the days,” and 3 = “nearly every day.” Sum scores were calculated and higher scores indicated greater severity of reported depressive symptoms. Internal consistency was acceptable (α = 0.70).

*Causal beliefs of mental illness* were assessed with a list of 18 possible causes (17). Participants were asked whether they believed a cause (e.g., “living in a big city”) could be responsible for their experienced complaints on a 5-point Likert scale ranging from 1 = “definitely is not a cause” to 5 = “definitely is a cause.”

To determine a *balanced biopsychosocial causal model,* we calculated the *BPS-CM* index. This index calculation was not explicitly mentioned in the study protocol and was developed during the analyses to operationalise a biopsychosocial vs. monocausal belief system (see primary research question no. 1 in the study protocol). The index score combined multiple, discreet causal beliefs while accounting for divergent agreement to different factors such as biogenetic, psychological, social, and environmental. Within-factor agreement was balanced with between-factor variance. In this way, it was possible to represent the participant’s personal causal belief model. A higher index score indicated a more heterogeneous and, therefore, balanced biopsychosocial belief system. Conceptually this meant that an individual with a higher score believed that their illness had multiple causal antecedents from different areas of life, e.g., biogenetic, childhood, and current social environment.

To determine the index we subsumed the 18 items into factors and then calculated the within-factor agreement and the between-factor variance, which we multiplied to consider the homogeneity of the belief system.

First, and similar to Stolzenburg et al. (15), different causal beliefs were subsumed into five factors: *childhood and upbringing* (e.g., “growing up in a broken family or in an institution”), *biomedical* (e.g., “heredity”), *social stress* (e.g., “occupational stresses and worries (including unemployment)”), *person-internal* (e.g., “weak willpower”), and *person-external* (e.g., “new phase of life, for example, retirement”).

Second, we recoded all items into a binary format, with 0 meaning “definitely is not a cause” and 1 meaning “definitely is a cause,” and calculated the within-factor agreement, with 0% representing the person’s belief that no item in any factor was seen as a possible cause, while 100% represented the belief that all items in all factors were seen as possible causes (each factor contributing 20% equally).

Third, we calculated the between-factor variance to consider the homogeneity of the belief system. The variance was calculated with factor mean scores. The higher inverted variance could be interpreted as higher similarity between factor means.

The *BPS-CM* index ranged from 2.12 to 42.80. The range was specific to the sample because it was dependent on the respective variance.

Correlation with the sum score across all items and the *BPS-CM* index was *r* = 0.68.

*Self-efficacy to self-help* was assessed with six items adapted from the BRAHMS-Study (37). Participants were asked to rate how certain they were that they could overcome specific barriers (e.g., “…if my complaints do not get better despite my efforts”) on a 5-point Likert scale ranging from 1 = “very uncertain” to 5 = “very certain.” Mean scores were calculated. Internal consistency was very good (α = 0.80).

*Self-efficacy to seek professional help* was assessed with seven items adapted from a study assessing the health care use of homeless people (38). Participants were asked to rate how confident they were that they could overcome barriers of the health care system (e.g., “I am able to deal with long waiting times”) on a 5-point Likert scale ranging from 1 = “not confident” to 5 = “very confident.” Mean scores were calculated. Internal consistency was very good (α = 0.86).

*Intention to seek help* was assessed with an adapted 15-item list of potential persons (e.g., psychotherapist) and institutions (e.g., counselling centre) on a 7-point Likert scale ranging from 1 = “extremely unlikely” to 7 = “extremely likely” (39).

For our analyses we constructed three different groups of professional help-seeking sources in line with previous studies (40, 41): g*eneral practitioners* (GPs), *mental health professionals* (i.e., psychologists, psychotherapists, and psychiatrists; MHPs), and *counselling* (i.e., counselling centre and social workers). The maximum score across the in-group items was taken to receive group indicators for a participant’s intention to seek help. Other sources of help-seeking, such as family, police, or a priest, were not included in the analyses.

*Help-seeking behaviour* was assessed with the same list of persons and institutions. The participants were asked if they sought help in the past 3 months (0 = “no,” 1 = “yes”). When stating “yes,” they were asked if it was due to their psychological complaints (1 = “yes, exclusively,” 2 = “yes, amongst other complaints,” 3 = “no, because of other complaints). The same three groups described for *intention* were used in these analyses. We collapsed responses for both follow-ups into dichotomous variables of help-seeking (0 = “did not seek help for their psychological complaints,” 1 = “sought help for their psychological complaints within 3 to 6 months after the baseline assessment”).

*Previous treatment experience* was assessed with the question “Have you ever received treatment for mental illness in your life?,” whereby multiple responses for different types of treatment were possible: “medical treatment,” “psychotherapy,” “art-, music-and/or sport-therapy,” “self-help groups,” “coaching and counselling,” and “online or telephone therapy.” We collapsed responses into a dichotomous variable of previous treatment experience (0 = has no experience, 1 = has treatment experience).

Additionally, *satisfaction with previous treatment* was assessed with the question “How content were you with your psychiatric or psychotherapeutic treatment in general?” with a 5-point Likert response scale ranging from 1 = “very discontent” to 5 = “content.”

Different instruments were used to assess the scope of stigmatising attitudes. Mean scores were calculated for the different attitudes. For a full account of all stigmatising attitudes assessed, refer to the study protocol (11).

*Perceived public stigma* and the *agreement thereof* were assessed with the short forms of the *SSMIS-public and-self* (42). Participants were asked to rate how much they agreed with statements concerning people with mental illnesses (e.g., “Most people with mental illness are dangerous”). The statements were primed with “I think the public believes…” (*awareness of stereotypes*) or “I think…” (*agreement to stereotypes*) and participants could rate their answers on a 5-point Likert scale ranging from 1 = “strongly disagree” to 5 = “strongly agree.” Internal consistency for the public-stigma subscale (α = 0.84) and self-stigma subscale (α = 0.80) were very good.

The tendency of a person to distance themselves socially from people living with mental illnesses was assessed with the German version of the *Social Distance Scale* (43). Participants were asked to rate how much they agreed with statements concerning people with mental illnesses (e.g., “How willing would you be about renting a room in your home to a person with severe mental illness?”) on a 5-point Likert scale ranging from 1 = “very unlikely” to 5 = “very likely.” Internal consistency was very good (α = 0.89).

The proclivity of *self-stigma for seeking out professional help* for one’s mental health complaints was assessed with the *SSOSH-SF* (44). Participants were asked to rate how much they agreed with statements (e.g., “I would feel inadequate if I went to a therapist for psychological help”) on a 5-point Likert scale ranging from 1 = “do not agree at all” to 5 = “agree completely.” Internal consistency was excellent (α = 0.92).

### 2.4 Blinding

The participants were blinded to the intervention. Outcome measures were self-reports by the participants. The data analyst was involved in the writing of the interventional material and the study design and was not blinded.

### 2.5 Statistical analysis and power analysis

First, sample characteristics and target and outcome variables were compared between the sub-samples of people with and without treatment experience; Pearson-Chi^2^ for categorical variables and Student’s t-test for continuous variables were used.

Second, we performed Pearson’s product–moment correlation analysis with the target, outcome, and control variables. Results were presented as bivariate correlations.

Third, to check if the intervention influenced the target variables, i.e., causal beliefs, and both self-efficacies, paired sample t-tests were performed. Benjamini-Hochberg value of p adjustment was used. The respective control groups were composed of the groups not receiving the interventional message of the target variable despite possibly receiving other interventional messages. The respective experimental groups were composed of the groups that received the targeted interventional message. For example, when analysing the effect of the causal belief intervention, groups 5–8, 13–16, and 21–24 were composed as the experimental group, and groups 1–4, 9–12, and 17–20 were defined as the control group (see Supplementary Table S2). This was possible due to the fractioned factorial design mentioned in section *2.2. Online intervention*.

Fourth, linear mixed model analyses between pre-intervention and post-intervention times were performed to investigate between-group effects (active control and intervention groups), allowing for possible interactions, when the participants received multiple intervention elements. This method was used to analyse the effect on both the target variables as well as help-seeking intention (for each intention group separately) accounting for the complex fractioned design. Bonferroni correction was used.

Fifth, we conducted multiple regressions (dependent variable: help-seeking intention) and logistic regressions (dependent variable: help-seeking behaviour) for the different intention/behaviour groups separately to analyse the predictive strength of different variables on a conceptual level. For the predictors, the post-intervention mean scores were used. Within the different regression analyses, interaction terms between the z-standardised *BPS-CM* index, *self-efficacy to self-help* score, and *self-efficacy to seek professional help* score were included, since it was likely that they would affect each other concerning help-seeking intention and behaviour. Additionally, we controlled for stigmatising attitudes, because there were substantial associations with both the target and outcome variables. For the multiple regression analyses, β-coefficients and corrected *R^2^* were reported. For logistic regressions, the adjusted Odds Ratios (*aOR*), and Pseudo-R^2^ as an approximation of explained variance (45) were provided. Interpretation of the effect coefficients was based on Cohens’ interpretation (46).

Concerning the power analysis for the different analyses, we estimated the needed sample size *a priori*: *N* = 1,084 for the t-tests (α = 0.05, 1-β = 0.95, *d* = 0.2), *N* = 1,077 for the correlation analyses [α = 0.05, 1-β = 0.95, ρ(H1) = 0.1, ρ(H0) = 0.0], *N* = 557 for the multiple regression analyses (α = 0.05, 1-β = 0.95, *f^2^* = 0.05, number of predictors = 14), and *N* = 988 [α = 0.05, 1-β = 0.95, OR = 1.3, Pr(Y = 1) H0 = 0.2]. All analyses were based on α = 0.05 and implemented in SPSS 28.

## 3 Results

### 3.1 Sample characteristics

The final sample size consisted of *N* = 1,368 individuals at baseline with *N* = 983 providing data on help-seeking behaviour within 3 to 6 months after baseline assessment. In Table 1 the descriptive statistics are presented and subsamples of participants with previous treatment experience are compared with the participants without any treatment experience. Missing data was detected for treatment experience at baseline (*n* = 54; compare with Table 1). No other missing scale values were detected within the relevant data. The total drop-out rate between the baseline assessment (*N* = 1,368) and the 3-month or 6-month follow-up (*N* = 983) was 28.14%. Potential reasons for attrition were analysed by conducting logistic regression analysis in which dropout was a dummy-coded outcome variable (1 = missing value due to drop-out at follow-up, 0 = no missing variable). This has previously been done by Beller et al. (47). The attrition analysis revealed that dropout within 3 months or 6 months was more likely when participants were younger, came from a bigger household, and had 9 years compared to 12 years of schooling.

**Table 1:** Baseline characteristics and help-seeking behaviours at follow-ups for the treatment experience sample compared to the no treatment experience sample with the significance of group comparison.

	Treatment experience [*n* (%) or *M* (*SD*)]	No treatment experience [*n* (%) or *M* (*SD*)]	Total sample [*n* (%) or *M* (*SD*)]
Baseline data	(*n* = 670)	(*n* = 644)	(*N* = 1,368)
Gender*			
Male	210 (31.34)	239 (37.11)	471 (34.40)
Female	460 (68.66)	405 (62.89%)	897 (65.60)
Age***	44.19 (14.55)	40.22 (15.65)	42.38 (15.22)
Depression severity***	13.01 (4.32)	11.87 (3.75)	12.50 (4.08)
Target variables			
Biopsychosocial causal beliefs***	22.50 (9.37)	20.07 (10.09)	21.38 (9.78)
Self-efficacy self-help	2.47 (0.57)	2.47 (0.55)	2.47 (0.56)
Self-efficacy seek help	3.05 (0.86)	3.01 (0.92)	3.03 (0.89)
Stigmatising attitudes			
Perceived public stigma	2.99 (1.00)	2.98 (0.94)	2.97 (0.97)
Stereotype agreement***	1.84 (0.70)	2.04 (0.75)	1.93 (0.73)
Social distance stigma***	2.26 (0.82)	2.48 (0.86)	2.35 (0.85)
Self-stigma of seeking help***	1.88 (0.96)	2.44 (1.06)	2.15 (1.05)
Help-seeking intention			
General practitioner (GP)***	3.08 (1.98)	2.33 (2.02)	2.71 (2.03)
Mental health professional (MHP)***	2.94 (2.09)	1.88 (2.00)	2.42 (2.11)
Counselling**	1.63 (1.88)	1.32 (1.77)	1.48 (1.83)
Follow-up data	(*n* = 491)	(*n* = 451)	(*N* = 983)
Help-seeking behaviour			
GP***	153 (31.16)	73 (16.19)	239 (24.30)
MHP***	73 (14.87)	37 (8.20)	119 (12.10)
Counselling*	33 (6.72)	17 (3.77)	52 (5.30)

Responses to different sources of help for follow-up data are not mutually exclusive. M, Mean; *SD*, standard deviation; Pearson-Chi2 for categorical variables and Student’s T-Test for continuous variables. *p < 0.05, **p < 0.01, ***p < 0.001.

In the supplementary material, the associations between the target variables (i.e., causal belief and both self-efficacy types), stigmatising attitudes, and different intention scores, as well as age and depression severity, are presented (Supplementary Table S3).

### 3.2 The interventional effect on the target variables

Table 2 shows interventional effects on causal beliefs and on both types of self-efficacies. For *causal beliefs*, post-mean scores were significantly higher than the pre-mean scores in both the control (*d* = 0.10) and the intervention (*d* = 0.13) groups. The pre-mean and post-mean scores for *self-efficacy to help oneself* did not differ significantly. The post-mean score for *self-efficacy to seek help* was significantly higher than the pre-mean score in the experimental group receiving an intervention (*d* = 0.14).

**Table 2:** T-test results for paired samples.

Intervention (*n*)	*M* pre	*M* post	*T (df)*	*d* [L-; U-CI]
Causal beliefs				
Control (692)	20.67	21.48	2.70 (691)	0.10** [0.03; 0.18]
Intervention (676)	20.34	21.27	3.25 (675)	0.13*** [0.05; 0.20]
Self-efficacy to self-help				
Control (681)	2.45	2.46	0.92 (680)	0.04 [−0.04; 0.11]
Intervention (687)	2.49	2.47	0.81 (686)	0.03 [−0.11; 0.04]
Self-efficacy to seek help^a^				
Control (461)	3.00	3.03	0.90 (460)	0.04 [−0.05; 0.13]
Intervention (449)	2.95	3.03	2.87 (448)	0.14** [0.04; 0.23]

Comparison of pre-mean and post-mean scores for the target variables themselves dependent on the interventional groups. *p < 0.05, **p < 0.01, ***p < 0.001. Benjamini-Hochberg value of *p* adjustment. ^a^For self-efficacy to seek help there were three groups (control, text, and video) but for these analyses only the text intervention was compared with the control group.

Additionally, to investigate the between-group interventional effects and possible interaction effects on the different target variables three linear mixed model analyses were executed.

The *BPS-CM* index score was negatively influenced if the participants received the *self-efficacy to self-help* intervention together with the *causal belief* intervention *F* (1, 1800.80) = 16.09 (*p* < 0.001) with a mean difference of-1.91 (*M_causal_* = 21.69, *M_interaction_* = 19.78, *p* < 0.01).

There were no significant between-group interventional or interactional effects on the two self-efficacy scores.

### 3.3 The interventional effect on help-seeking intention

Multiple linear mixed model analyses were conducted for the different help-seeking intention groups (i.e., GP, MHP, counselling, and informal). The main and interaction terms of the interventions were included in the model.

Concerning interventional effects on the intention to seek help from a GP, the intervention intended to strengthen *self-efficacy to self-help* positively influenced intention with *F* (1, 1811.51) = 5.60 (*p* = 0.02) with a mean difference of 0.23 (*M_self-help_* = 2.82, *M_control_* = 2.59, *p* = 0.02). The intervention intended to strengthen *self-efficacy to seek help* positively influenced intention with *F* (1, 1811.51) = 6.50 (*p* = 0.01) with a mean difference of 0.24 (*M_seek-help_* = 2.82, *M_control_* = 2.58, *p* = 0.01).

Concerning interventional effects on the intention to seek help from an MHP, the intervention intended to strengthen *self-efficacy to self-help* positively influenced intention with *F* (1, 1811.90) = 4.85 (*p* = 0.03) with a mean difference of 0.22 (*M_self-help_* = 2.56, *M_control_* = 2.35, *p* = 0.03). The intervention intended to strengthen *self-efficacy to seek help* positively influenced intention with *F* (1, 1811.90) = 4.45 (*p* < 0.04) with a mean difference of 0.21 (*M_seek-help_* = 2.56, *M_control_* = 2.35, *p* = 0.01).

There were no significant main effects for the intentions to seek help from a counselling centre and social worker nor friends and family.

Furthermore, receiving the intervention for *causal beliefs*, *self-efficacy to self-help*, and *self-efficacy to seek help* had a significant, positive effect on the intention to seek help from a GP with *F* (1,1811.51) = 9.82, *p* < 0.01, as well as from an MHP, *F* (1,1811.90) = 11.86, *p* < 0.001.

### 3.4 Prediction of help-seeking by causal beliefs and self-efficacy beliefs

To analyse what influences help-seeking on a conceptual level, linear multiple regression analyses were performed with intention as the dependent variable and logistic multiple regression with behaviour after 3 or 6 months as the dependent variable. Results are reported in Tables 3, 4.

**Table 3:** Beta coefficients of linear multiple regression models predicting *help-seeking intention* for mental health complaints from general practitioners, mental health professionals, and counselling sources by causal beliefs, self-efficacy to self-help, and to seek professional help (as continuous predictors) in a sample of adults with depressive complaints (*N* = 1,368).

	General practitioner			Mental health professional			Counselling		
	β	95% CI for B		β	95% CI for B		β	95% CI for B	
		Lower	Upper		Lower	Upper		Lower	Upper
Causal beliefs	0.09^**^	0.01	0.03	0.12^***^	0.01	0.04	0.23^***^	0.03	0.06
Self-efficacy to self-help	−0.00	−0.23	0.22	0.04	−0.08	0.38	0.07^*^	0.03	0.43
Self-efficacy to seek help	0.25^***^	0.42	0.72	0.17^***^	0.25	0.56	0.16^***^	0.19	0.46
Causal*seek help interaction	−0.01	−0.15	0.09	0.00	−0.12	0.12	−0.02	−0.14	0.08
Self-help*seek help interaction	−0.03	−0.15	0.05	−0.10^***^	−0.28	−0.08	−0.05	−0.16	0.02
Causal*self-help*seek help int.	0.02	−0.06	0.11	−0.02	−0.12	0.06	−0.04	−0.13	0.03
*Corrected R^2^ (model 1)*	*0.07*			*0.07*			*0.09*		
Perceived public stigma	−0.06^*^	−0.24	−0.01	−0.03	−0.19	0.05	−0.05	−0.21	0.01
Stereotype agreement	−0.00	−0.19	0.18	0.05	−0.03	0.34	0.09^**^	0.05	0.39
Social distance stigma	0.02	−0.11	0.19	−0.04	−0.25	0.05	−0.06	−0.26	0.01
Self-stigma of seeking help	−0.07^*^	−0.25	−0.01	−0.15^***^	−0.43	−0.18	−0.04	−0.18	0.04
*Corrected R^2^ (model 2)*	*0.09*			*0.10*			*0.09*		
Depression severity	0.03	−0.01	0.04	0.09^**^	0.02	0.08	0.02	−0.02	0.04
Age	0.13^***^	0.01	0.03	−0.03	−0.01	0.01	−0.02	−0.01	0.01
Gender (men)^a^	−0.03	−0.36	0.12	−0.04	−0.40	0.10	−0.06^*^	−0.46	−0.02
Treatment experience (none)^a^	0.14^***^	0.32	0.79	0.20^**^	0.59	1.07	0.05	−0.01	0.42
*Corrected R^2^ (model 3)*	*0.14*			*0.15*			*0.10*		

β, Beta coefficients; 95% CI, 95% confidence interval for unstandardized B coefficients. ^a^Reference categories in brackets. It is controlled for income, school education, and higher education. The coefficients are not reported in detail because there are no systematic influences (except for a negative influence of higher school education compared to “9 years in school” and help-seeking intention from an MHP; *β* = −0.10 to −0.15). *p < 0.05, **p < 0.01, ***p < 0.001.

**Table 4:** Adjusted odds ratios of logistic regression models predicting *help-seeking behaviour* for mental health complaints from general practitioners, mental health professionals, and counselling sources by help-seeking intention, causal beliefs, self-efficacy to self-help, and to seek professional help (as continuous predictors) in a sample of adults with depressive complaints (*n* = 983).

	General practitioner			Mental health professional			Counselling		
	aOR	95% CI		aOR	95% CI		aOR	95% CI	
		Lower	Upper		Lower	Upper		Lower	Upper
Intention (respectively)	1.68^***^	1.51	1.87	1.28^***^	1.14	1.44	1.61^***^	1.36	1.92
*R^2^ (model 1)*	*0.22*			*0.08*			*0.12*		
Causal beliefs	1.01	0.98	1.03	1.01	0.98	1.03	1.01	0.97	1.05
Self-efficacy to self-help	0.97	0.66	1.42	0.70	0.43	1.14	0.99	0.49	1.99
Self-efficacy to seek help	0.97	0.74	1.26	1.28	0.92	1.77	0.96	0.60	1.53
Causal*seek help interaction	0.88	0.71	1.08	0.94	0.74	1.21	1.17	0.82	1.66
Self-help*seek help interaction	0.82^*^	0.68	0.99	1.04	0.85	1.28	1.03	0.77	1.38
Causal*self-help*seek help int.	0.80^**^	0.68	0.94	0.93	0.78	1.11	0.93	0.71	1.22
*R^2^ (model 2)*	*0.24*			*0.09*			*0.13*		
Perceived public stigma	0.92	0.76	1.12	0.94	0.74	1.18	1.26	0.91	1.73
Stereotype agreement	0.97	0.71	1.34	1.02	0.70	1.48	0.76	0.45	1.28
Social distance stigma	0.60^***^	0.45	0.78	0.92	0.67	1.26	1.16	0.73	1.83
Self-stigma of seeking help	1.11	0.90	1.36	0.85	0.65	1.11	0.99	0.68	1.44
*R^2^ (model 3)*	*0.27*			*0.10*			*0.14*		
Depression severity	1.09^***^	1.04	1.15	1.12^***^	1.06	1.18	1.13^**^	1.04	1.23
Age	1.02^*^	1.00	1.04	1.00	0.98	1.02	1.01	0.98	1.04
Gender (men)^a^	1.16	0.77	1.74	1.02	0.62	1.70	0.83	0.40	1.71
Treatment experience (none)^a^	1.61^*^	1.07	2.43	1.38	0.83	2.28	1.29	0.62	2.69
*R^2^ (model 4)*	*0.35*			*0.16*			*0.21*		

aOR, adjusted Odds Ratio; 95% CI, 95% confidence interval for aOR; *R^2^*, Nagelkerke’s *R^2^.* It is controlled for income, school education, and higher education. The coefficients are not reported in detail because there are no systematic influences. ^a^*R*eference categories in brackets. *p < 0.05, **p < 0.01, ***p < 0.001.

### 3.5 Prediction of help-seeking by satisfaction with previous treatment

To analyse the predictive value of *satisfaction with previous treatment* on help-seeking, the same regression analyses were performed as shown in Tables 3, 4 (see section *3.4. Prediction of help-seeking by causal beliefs and self-efficacy beliefs*), with the difference that treatment satisfaction could only be assessed in the subsample of participants who had past experiences (*n* = 670). The same methods and control variables were used.

Concerning help-seeking intention: *satisfaction with previous treatment* significantly predicted intention to see a GP (β = 0.19 [0.16; 0.39], *ΔR^2^* = 0.07, *p* < 0.001), intention to see an MHP (β = 0.17 [0.14; 0.39], *ΔR^2^* = 0.05, *p* < 0.001), and intention to seek help from a counselling centre/counsellor (β = 0.08 [0.00; 0.22], *ΔR^2^* = 0.02, *p* = 0.002).

Concerning help-seeking behaviour: *satisfaction with previous treatment* did not significantly predict any help-seeking behaviour.

## 4 Discussion

In this article, we examined how causal and self-efficacy beliefs facilitate help-seeking intention and behaviour, both through interventional manipulation and by analysing their conceptual associations. Specifically, how and in what ways help-seeking is influenced by causal beliefs and self-efficacy beliefs and how these beliefs interact with each other. We also examined how previous treatment experience and the satisfaction therewith influenced the different process variables.

*Does a balanced biopsychosocial causal model of depression positively influence help-seeking intention?* The intervention on a biopsychosocial causal belief model had no significant effect on help-seeking intention, neither by itself nor compared to the control groups. However, a significant effect on intention emerged when including interaction terms for both self-efficacy interventions. While this indicates that this specific intervention did not have an effect on the biopsychosocial diversity of a person’s causal beliefs or on the intention to seek professional help, it also points to the potential of more complex interventions that consider additional aspects, such as self-efficacy. It should be noted that the control groups were active controls that received a similar combination of other interventions [e.g., depression literacy, which is conceptually closely related to causal beliefs; (40)] even if they did not receive the causal belief interventional message. Additionally, participation in such a study could have heightened awareness of one’s own symptoms and started reflection processes irrespective of group allocation.

However, on a conceptual level, there was a clear association between a more heterogeneous belief system and the intention to seek help from a professional, as indicated by significant correlations (see Supplementary Table S3) and regression weights. Interestingly, these associations were stronger for the intention to seek help in a counselling centre or from a social worker than from a GP or an MHP. Because the *BPS-CM* index measures the heterogeneity of a person’s causal beliefs, those with higher scores were more likely to believe that biological, psychological, and social causal antecedents could be responsible for their issues. As the literature shows, this leads to reduced help-seeking from GPs and MHPs (12, 13), possibly explaining why the associations were weaker for these more formal health care sources. Furthermore, this could lead to an openness to seek help from a source outside of the health care system, explaining the predictive value of β = 0.23 with the intention to seek help in a counselling centre or from a social worker. The associations remained significant even in the context of other predictors, indicating a robust finding. The research question was answered insofar as that a more balanced biopsychosocial causal belief system positively predicts help-seeking intention. However, the experimental manipulation did not work as planned, i.e., compared with the active controls.

*Does a higher self-efficacy to self-help negatively influence help-seeking intention?* The intervention in self-help strategies had a small, positive effect on help-seeking intention (both from a GP and from MHPs). This was surprising and counter-hypothetical. However, even though the items had a very good internal consistency they were not psychometrically validated and their sensitivity to change was unknown. On the other hand, the measure correlated strongly (*r* = 0.48) with the second self-efficacy measure used in this study, indicating construct validity. In addition, the measure correlated negatively with depression severity, which was also in line with self-efficacy literature (19, 25). This taken together pointed to a robust measure and, therefore, indicated that the intervention did in fact have an effect on the intention to seek help even if not on the efficacy beliefs themselves.

Additionally, on a conceptual level, there were clear positive associations between self-efficacy to self-help and different intentions to seek help, which again contradicted the research question. However, the correlations (see Supplementary Table S3) were small and, when considered in the context of other predictors, the values were non-significant (with the exception of β = 0.07 on intention to seek help in a counselling centre or from a social worker). Interestingly, the interaction term between both self-efficacy beliefs had a negative predictive value β = −0.10 on the intention to seek help from an MHP. After considering this, the same multiple regression analyses were conducted, but this time separately for three levels of depression severity, namely, *mild* (PHQ-9 score 8 to 10), *moderate* (PHQ-9 score 11 to 15), and *moderately severe/severe* (PHQ-9 score 16 to 27), according to Kroenke et al. (48). The results of the multiple and logistic regressions can be seen in the supplementary material (Supplementary Table S4). The interaction effect disappeared in the groups with moderate or moderately severe/severe depression and was only significant in the group with mild depression (β = −0.17). Self-efficacy to seek help became increasingly important with greater symptom severity (mild: β = 0.14, moderate: β = 0.16; moderately severe/severe: β = 0.27). We interpreted these findings to indicate that self-efficacy to self-help is more relevant for participants with mild depressive symptoms and will inhibit help-seeking intention, which is plausible, when considering that one of the largest reported treatment seeking barriers is wanting to handle the problem on one’s own (10). With increased symptom severity, self-efficacy to self-help loses relevance and self-efficacy to seek help becomes increasingly relevant, assumedly because the need for help becomes more pressing with higher illness severity. However, because the analyses were explorative and not confirmative, further research is needed to elucidate these interactional findings of how self-efficacy influences help-seeking intention. The hypothesis was rejected.

*Does a higher self-efficacy to seek help positively influence help-seeking intention and does it make help-seeking behaviour more likely?* The intervention on help-seeking strategies had a small, positive effect on help-seeking intention (both from a GP and MHPs). It had a significant effect on its target variable self-efficacy to seek help (*d* = 0.14), and when participants received all three intervention elements there was a small significant effect on intention to seek help (both from a GP and MHPs). Especially considering that the other two interventions (i.e., on causal beliefs and self-efficacy self-help) did not influence their intended target variable it is likely that the intervention designed to influence self-efficacy to seek help is the main contributor to the effect on intention. In addition, the interventions were very short (*M_word count_* = 170) and were administered online. Because of the online setting and the short and informational character of the intervention we only expected small effect sizes (11). Taking this into account, the results indicated that even a very low threshold, namely, an online intervention designed to *strengthen self-efficacy to seek help*, especially in combination with additional informational messages, such as biopsychosocial causal beliefs and vulnerability-stress, can strengthen self-efficacy beliefs and support a person in forming their intention to seek help from a GP or MHP.

On a conceptual level, there were clear positive associations between self-efficacy to seek help and different intentions to seek help (see Supplementary Table S3). These correlations remained significant predictors when considered in the context of other variables such as self-efficacy to self-help or stigmatising attitudes. Therefore, the research question was answered insofar as self-efficacy to seek help positively predicted help-seeking intention.

On the other hand, help-seeking behaviour was not predicted by self-efficacy to seek help, the main predictor being the respective intention to seek professional help, as has been discussed in current research on the “intention-behaviour gap” (23, 31). It could also be that people with depressive complaints not only suffer from reduced self-efficacy (49) but that when seeking professional help other factors such as perceived accessibility to the health care systems are of greater importance than their own self-efficacy beliefs.

The interaction effects for different levels of depression severity (see Supplementary Table S5) were difficult to interpret because there were no main effects, which is why further research is needed to explore possible negative effects self-efficacy might have on actual help-seeking behaviour. The hypothesis was, therefore, rejected concerning actual behaviour but could be accepted concerning help-seeking intention.

*Is treatment experience positively associated with both self-efficacy beliefs, as well as help-seeking intention and behaviour? In addition, how is satisfaction with previous treatment associated with these variables?* The results differed depending on whether treatment experience as such was examined or the satisfaction therewith.

Concerning self-efficacy, treatment experience did not seem to influence self-efficacy beliefs regarding self-help or seeking help. There was no significant difference between the two groups (see Table 1). However, the significant, positive correlations of satisfaction with previous treatment experience with both self-efficacy beliefs indicated that there was a subjectively felt quality of therapy that influenced self-efficacy (see Supplementary Table S3). Possibly, higher satisfaction with previous treatment reflected better therapeutic results, which in turn showed itself in higher self-efficacy beliefs.

Concerning help-seeking intention, the findings clearly indicated that treatment experience was an important factor. People were more likely to have a higher intention to seek help for their current complaints if they had done so before. The findings remained significant even in the context of other variables influencing intention.

Concerning behaviour, the findings were mixed. People with treatment experience were more likely to seek help from a professional, especially a GP, than if they had no previous treatment experience. Satisfaction with previous treatment, however, did not influence behaviour. This indicates that it is the processes of help-seeking, e.g., knowing where to go, what the doctor will ask, what to expect, and less the quality of previous experiences, i.e., satisfaction, that seems to have an influence on help-seeking behaviour. We suggest that it is important, then, to support people in their behaviour by strengthening their knowledge of how to seek help and what structural barriers to expect. In addition, the possibility of simulation interventions, e.g., using virtual reality technology, could train participants to overcome actual barriers when seeking help.

There are some important strengths and limitations that should be highlighted. The study was preregistered (11) and the aims and procedure were transparently documented in the study protocol. The drop-out rate between the baseline assessment (*N* = 1,368) and both the 3-month or 6-month follow-ups together (*N* = 983) was acceptable with 28.14% and there were only a few missing data for treatment experience (3.95%). The data control procedure was very high, especially our criteria to only include participants with a stable PHQ-9 score over the first 36 h (baseline and intervention assessment) and to exclude all participants who finished the study too quickly. We did this to counteract the problem that in online settings outside distractions are more probable than in a laboratory setting, even if the level of attention does not seem to be impaired (50). Overall, however, the online setting was a strength. First, the sample was more representative since we were able to reach people across Germany. Second, it seemed likely that people with current depressive symptoms who were currently not seeking help would be more willing to participate in a low-threshold online study than a full clinical trial. However, one drawback was that the participants had to be registered in an online panel and it was, therefore, likely that our population was more motivated to participate in surveys and were younger than the general population. Another issue was that drop-out was more likely when a person was younger, came from a multi-person household, and was less educated; wherefore, there was a bias in our study and possibly the intervention material, which seemed to be implicitly preferred by people with higher education living in more individualistic households.

The study controls were active control groups, with all participants receiving at least a vignette describing a person with depressive complaints and most receiving some type of intervention. The vignette alone likely triggered a reflective process on their current complaints. Therefore, on the one hand, the intervention effects discussed in this article can be seen as very robust. Possibly, some effects stayed undetected due to the complexity of the fractioned factorial design. On the other hand, the fractioned design might have led to a confounding of the main effects. Subsequent research could then analyse the factors more insularly, now that the positive tendency has been established.

## 5 Conclusion

In conclusion, the results show promising interventional effects strengthening self-efficacy to seek help. Less clear are the effects of the interventions concerning help-seeking intention. Therefore, we think that an intervention would be effective if designed to strengthen self-efficacy beliefs as well as causal beliefs. Furthermore, the person should be encouraged to reflect on both how and when to manage their own symptoms, for example, when their symptoms are still mild, and when it would be better to seek professional health care. This seems a sensible approach when considering the conceptual associations and especially the interaction effects found concerning the intention to seek help dependent on depression severity levels. In addition, when considering the effect that previous treatment has on help-seeking behaviour, interventions should include concrete suggestions of how to actually seek help and which structural barriers have to be overcome in the process. This could reduce the potential reach of such an intervention, making it less generalisable and more local than the current study.

Questions relating to the exact interactions of self-efficacy and causal beliefs on help-seeking remained unanswered and should be considered in future studies examining the help-seeking process of people with depressive complaints.

## Data availability statement

The raw data supporting the conclusions of this article will be made available by the authors, without undue reservation. The intervention material is available in the supplement of the study protocol (11).

## Ethics statement

The studies involving humans were approved by Ethics Commission of the University Medicine Greifswald (BB 061/18). The studies were conducted in accordance with the local legislation and institutional requirements. The participants provided their written informed consent to participate in this study.

## Author contributions

TM developed and drafted the manuscript, and analysed and interpreted the data. TM and L-JP contributed to the design of the study and the acquisition of the data. ST and HM contributed to the conception of the work. L-JP, ST, HM, SS and GS revised the draft. GS and SS managed the project. All authors contributed to the article and approved the submitted version.
