# Supplementary material for: The effects of causal and self-efficacy beliefs on help-seeking for people with depressive complaints: a quasi-experimental online study
Source: Front Psychiatry. 2023 Nov 30;14:1232848. doi: 10.3389/fpsyt.2023.1232848 (PMC10720663; doi:10.3389/fpsyt.2023.1232848)
Supplement: Supplementary file 1 [file Data_Sheet_1.pdf]

Supplementary Material to the article:

McLaren et al. (2023). The effects of causal and self-efficacy beliefs on help-seeking for people with depressive complaints: a quasi-experimental online study. *Front. Psychiatry*. DOI: 10.3389/fpsyt.2023.1232848

Content

In this supplement the participant flow-diagram as well as further result tables are presented. First, the flow diagram (S1). Second, the systematically determined groups of the fractioned factorial design (S2). Third, a correlation matrix of all relevant variables (S3) and fourth, additional results of further multiple regression analyses (S4 & S5).

S4 & S5: The same multiple regression analyses were conducted as in *3.4. Prediction of help-seeking by causal beliefs and self-efficacy beliefs*, but this time separately for three levels of depression severity: *mild* (PHQ-9 score 8 to 10), *moderate* (PHQ-9 score 11 to 15), and *moderately severe/severe* (PHQ-9 score 16 to 27). The groups are according to Kroenke et al. (2001). The results are discussed in the main paper.

Figure S1

Participant flow diagram adapted from the CONSORT guidelines. Allocations to the different groups (1-24) as well as drop-outs and exclusion criteria reported.

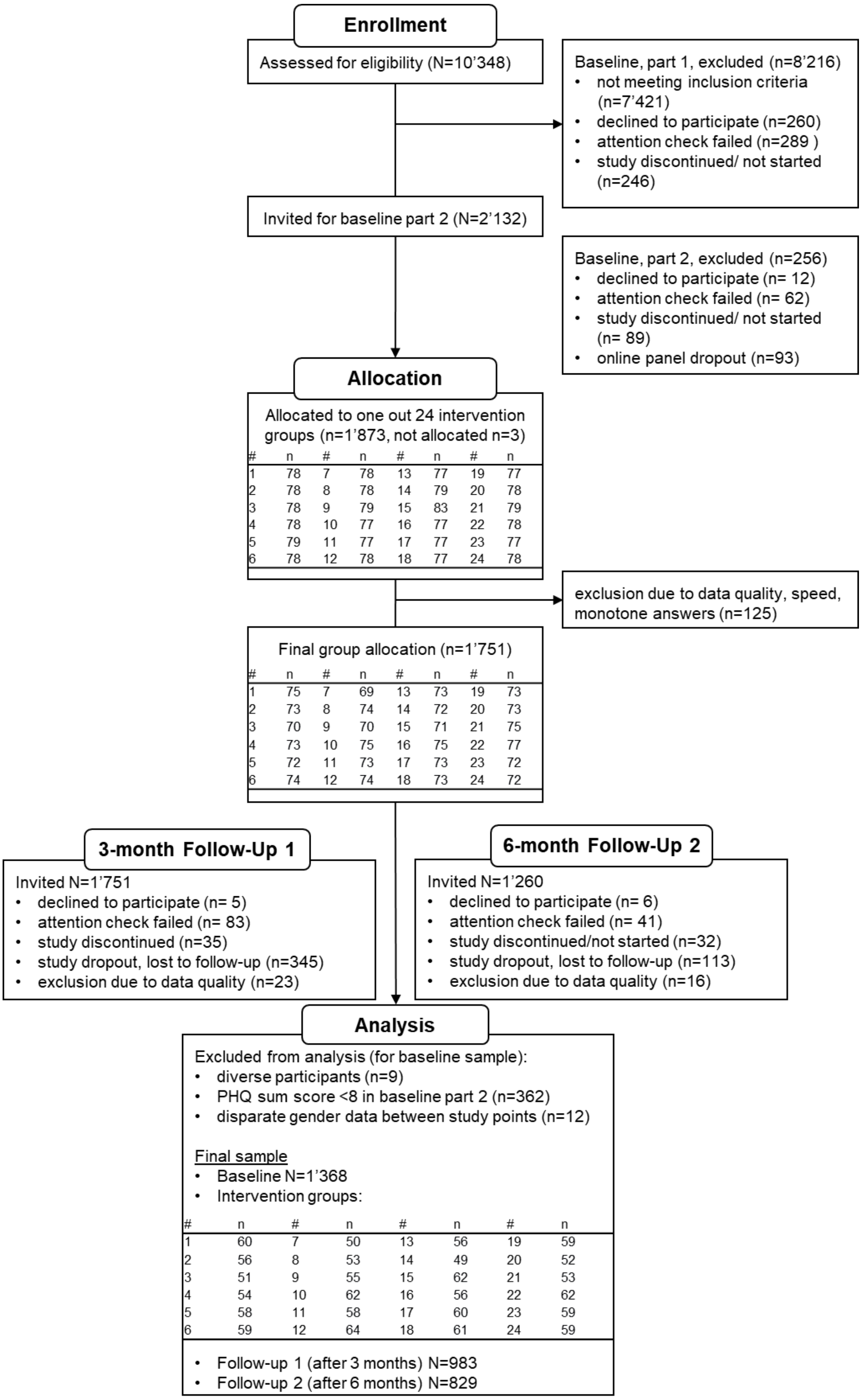

Table S2

Fractional factorial design with 24 groups

Group #	Interventional messages: Continuum Belief	Mental Health Literacy	Causal Belief	Self-Efficacy (Self-Help)	Self-Efficacy (Seek Help)	Exp. Group[b]
1[a]						
2				T	T	Self, Seek
3		T			T	Seek
4		T		T	V	Self
5			T		T	CB, Seek
6			T	T	V	CB, Self
7		T	T		V	CB
8		T	T	T		CB, Self
9	T				T	Seek
10	T			T	V	Self
11	T	T			V	
12	T	T		T		Self,
13	T		T		V	CB
14	T		T	T		CB, Self
15	T	T	T			CB
16	T	T	T	T	T	CB, Self, Seek
17	V				V	
18	V			T		Self
19	V	T				
20	V	T		T	T	Self, Seek
21	V		T			CB
22	V		T	T	T	CB, Self, Seek
23	V	T	T		T	CB, Seek
24	V	T	T	T	V	CB, Self

Note. T = text-based intervention, V = video-based intervention. Information for both intervention-types are the same.

This design has a resolution of V and can therefore statistically differentiate between main and two-wise interaction effects.

[a]. Group #1 receives no further interventional material.

[b]. The experimental groups are composed following fractional factorial design; symbolised as CB (Causal Beliefs), Self (Self-Efficacy to Self-Help), and Seek (Self-Efficacy to Seek Help). The respective control groups are composed from the groups in which the respective interventional message was not presented.

Table S3

Correlation matrix for all relevant variables

	1	2	3	4	5	6	7	8	9	10	11	12	13
1. age	–												
2. depression severity	-.06*	–											
3. BPS-CM index[a]	-.05	.08**	–										
4. self-efficacy to self-help	.02	-.15**	-.07**	–									
5. self-efficacy to seek help	.03	-.24**	-.06*	.48**	–								
<u>stigmatising attitudes</u>													
6. perceived public stigma[b]	-.15**	.09**	.06*	-.08**	-.05	–							
7. stereotype agreement[c]	-.08**	.01	.21**	-.07**	-.08**	.23**	–						
8. social distance stigma[d]	.16**	-.08**	.04	-.06*	-.08**	.03	.46**	–					
9. self-stigma of seeking help[e]	-.01	.07**	.05	-.17**	-.27**	.09**	.34**	.28**	–				
<u>intention to seek help</u>													
10. general practitioner	.21**	-.03	.08**	.12**	.26**	-.08**	-.05	.00	-.16**	–			
11. mental health professional	.05	.05	.13**	.13**	.22**	-.02	-.02	-.08**	-.22**	.63**	–		
12. counselling (centre)	.01	-.00	.20**	.13**	.21**	-.03	.06*	-.04	-.08**	.47**	.58**	–	
13. satisfaction[f]	.18**	-.12**	.01	.18**	.27**	-.03	-.15**	-.10*	-.16**	.27**	.24**	.14**	–

Note. Pearson correlation coefficients. * $p < .05$, ** $p < .01$

[a] causal belief index representing a balanced BioPsychoSocial Causal Model

[b] measured with the Short-Form of the Self-Stigma of Mental Illness Questionnaire – Public

[c] measured with the Short-Form of the Self-Stigma of Mental Illness Questionnaire – Self

[d] measured with the Social Distance Scale

[e] measured with the Short-Form of the Self-Stigma for Seeking Help Questionnaire

[f] satisfaction with previous treatment experience

S4, “Table 4, addendum”

Beta coefficients of linear multiple regression models predicting *help-seeking intention* for mental health complaints from a *mental health professional* by causal beliefs, self-efficacy to self-help, and self-efficacy to seek professional help. The sample of adults with depressive complaints is stratified into three groups representing mild (8-10), moderate (10-15), and moderately severe to severe depression (16-27) according to Kroenke et al., (2001). N = 1368

	mild	moderate	moderately severe to severe
	(n = 557)	(n = 515)	(n = 296)
causal beliefs	.10*	.15**	.07
self-efficacy to self-help	.01	.02	.10
self-efficacy to seek help	.14**	.16**	.27***
causal*seek help interaction	.05	-.01	-.08
self-help*seek help interaction	-.17***	.01	-.11
causal*self-help*seek help interaction	-.07	-.02	.06
perceived public stigma	-.03	-.04	-.01
stereotype agreement	.11*	.05	-.02
social distance stigma	-.11*	.03	-.06
self-stigma of seeking help	-.14**	-.16**	-.14*
corrected R^2	*.15*	*.09*	*.23*

Note. Controlled for age, gender, treatment experience, income level, school education, and higher education; for instruments/ scales used, refer to the main article

* $p < .05$, ** $p < .01$, *** $p < .001$

S5, “Table 5, addendum”

Adjusted odds ratios of logistic regression models *predicting help-seeking behaviour* for mental health complaints from *general practitioner* by help-seeking intention, causal beliefs, self-efficacy to self-help, and to seek professional. The sample of adults with depressive complaints is stratified into three groups representing mild (8-10), moderate (10-15), and moderately severe to severe depression (16-27) according to Kroenke et al., (2001). *N* = 983

	mild	moderate	moderately severe to severe
	(n = 409)	(n = 351)	(n = 203)
intention	1.80***	1.77***	1.89***
causal beliefs	1.02	1.01	0.98
self-efficacy to self-help	0.58	1.35	1.06
self-efficacy to seek help	1.33	0.81	0.66
causal*seek help interaction	1.03	0.68	0.67
self-help*seek help interaction	1.02	0.41***	0.89
causal*self-help*seek help interaction	0.90	0.63*	0.66*
perceived public stigma	0.82	1.46*	0.57*
stereotype agreement	1.21	0.73	1.59
social distance stigma	0.47***	0.66	0.55
self-stigma of seeking help	0.87	1.09	1.23
Nagelkerke's R^2	*.38*	*.40*	*.60*

Note. Controlled for age, gender, treatment experience, income level, school education, and higher education; for instruments/ scales used, refer to the main article

* $p < .05$, ** $p < .01$, *** $p < .001$

References

Kroenke, K., Spitzer, R. L., & Williams, J. B. (2001). The PHQ-9: Validity of a brief depression severity measure. *Journal of General Internal Medicine*, *16*(9), 606–613. https://doi.org/10.1046/j.1525-1497.2001.016009606.x
